# Functional profile of host microbiome indicates *Clostridioides difficile* infection

**DOI:** 10.1080/19490976.2022.2135963

**Published:** 2022-10-26

**Authors:** Etienne Nzabarushimana, Haixu Tang

**Affiliations:** a Luddy School of Informatics, Computing and Engineering, Indiana University, Bloomington, Indiana, USA; b Division of Gastroenterology, Massachusetts General Hospital, Boston, Massachusetts, USA

**Keywords:** Functional profile, metagenomics, microbiome, *Clostridioides difficile* infection, logistic regression, machine learning, antibiotics, fecal microbiota transplantation, antibiotic impact

## Abstract

Clostridioides difficile infection (CDI) is a gastro-intestinal (GI) infection that illustrates how perturbations in symbiotic host–microbiome interactions render the GI tract vulnerable to the opportunistic pathogens. CDI also serves as an example of how such perturbations could be reversed via gut microbiota modulation mechanisms, especially fecal microbiota transplantation (FMT). However, microbiome-mediated diagnosis of CDI remains understudied. Here, we evaluated the diagnostic capabilities of the fecal microbiome on the prediction of CDI. We used the metagenomic sequencing data from ten previous studies, encompassing those acquired from CDI patients treated by FMT, CDI-negative patients presenting other intestinal health conditions, and healthy volunteers taking antibiotics. We designed a hybrid species/function profiling approach that determines the abundances of microbial species in the community contributing to its functional profile. These functionally informed taxonomic profiles were then used for classification of the microbial samples. We used logistic regression (LR) models using these features, which showed high prediction accuracy (with an average AUC≥0.91), substantiating that the species/function composition of the gut microbiome has a robust diagnostic prediction of CDI. We further assessed the confounding impact of antibiotic therapy on CDI prediction and found that it is distinguishable from the CDI impact. Finally, we devised a log-odds score computed from the output of the LR models to quantify the likelihood of CDI in a gut microbiome sample and applied it to evaluating the effectiveness of FMT based on post-FMT microbiome samples. The results showed that the gut microbiome of patients exhibited a gradual but steady improvement after receiving successful FMT, indicating the restoration of the normal microbiome functions.

## Introduction

Gut microbiome is inextricably linked to human health and well-being. A normal human gut microbiome lives in a symbiotic relationship with the host and usually exhibits a rich diversity in its taxonomic profile. The role and the impact of gut microbiome on human health have been gradually uncovered in the past decade.^1–5^ Scientific and experimental evidence substantiates that enteric microorganisms play a role in maintaining the gut physiologic homeostasis by offering critical metabolic functions, colonization resistance against harmful pathogens, protection of the intestinal barrier and modulating immune responses of the host.^1^ Growing evidence also suggests a bidirectional communication between the central nervous system and the host gut microbiome.^6^ This gut microbiome–host interplay is facilitated by very intricate and complex interactions marked by host–microbe feedback, as well as microbe–microbe crosstalk. These interactions are driven by various biochemical reactions which facilitate the response and adaptation of microorganisms to environmental changes such as diet, host lifestyle, antibiotic usage, etc.^7^ It is not a trivial task to understand the nature of this microbiome–host partnership.

Compositional profiling of the gut microbiome from healthy humans indicates a high microbial diversity across individuals, potentially due to the complex behaviors of the associated-hosts.^8^ The heterogeneity is even more pronounced in human subjects with altered gut microbiomes.^9^ The widely reported host-to-host variance in gut microbiome taxonomic composition has significant implications. First, the gut microbiome does not function as a collection of individual species or strains but rather as a dynamic collective ecosystem that promotes, facilitates, and modulates the context-dependent microbiome functions.^10^ Second, the gut microbiome is characterized by a high functional redundancy such that species could become interchangeable in the life-cycle of a microbiome without changing the microbiome integrity and impact.^11,12^ Regrettably, the ability to determine every member of the microbial community remains nearly impossible. Additionally, analytical tests and quantitative methods to establish the underlying mechanistic understanding of the relationship between the functions, the evolution, and the interactions of microbial communities and the host phenotype (healthy or diseased) or a particular biological process remain also largely limited.

Large-scale human microbiome initiatives^13,14^ have generated massive omic data from patients with various clinical conditions such as inflammatory bowel diseases,^15^ irritable bowel syndrome,^16^ CDI,^17–19^ as well as healthy controls. These studies and many others^20–22^ have enabled the study of functional and compositional changes observed in gut microbiome of healthy and diseased individuals. Among these clinical conditions, CDI is relatively well studied and illustrates how alterations in equipoised host–microbiome interactions render the human GI tract vulnerable to pathogenic attacks.^23–25^

CDI is caused by the infection of *C. difficile*, a toxin-producing bacteria and an opportunistic pathogen residing in the human GI tract. *C. difficile* is easily transmissible in healthcare settings, and its spores are metabolically dormant and resistant to standard disinfectants.^26^ However, once ingested, they germinate in response to environmental cues such as certain bile salts and amino acid germinants, though the process and the factors regulating its germination pathways are not mechanistically and decisively elucidated.^27–29^ The risk of getting CDI increases with prolonged stay in healthcare settings (such as hospitals and nursing homes) and the use of antibiotics.^30^ In fact, CDI is particularly severe in the elderly population (aged 65 and older), resulting in a high mortality rate within the first month of CDI diagnosis.^31^ Clinical diagnosis of CDI traditionally involves noninvasive examinations based on relevant clinical manifestations including diarrhea, presence of toxins or detection of toxin-producing *C. difficile* strains through stool tests or by nucleic acid amplification tests (NAAT).^32^ CDI diagnosis could also involve imaging tests of the colon or invasive diagnostics such as colonoscopy.

However, due to the complexity of factors associated with CDI pathogenesis and the range of its clinical manifestations,^33^ the currently used tests may lead to conflicting diagnostic indications.^34^ Accurate diagnostic approaches that take into account the gut microbiome impact on *C. difficile* colonization are needed and could lead to better prevention or therapeutic tools and options.^35^ Numerous studies have revealed pathogenic mechanisms associated with CDI and indicated that both the *C. difficile* toxin mechanism of action (along with its virulence-associated pathways) and the non-virulence factors are equally important in effective characterization of CDI.^34,36–40^

In this paper, we investigated the approach of applying machine learning to CDI prediction using fecal microbiome from CDI patients, in an attempt to identify the microbial features associated with CDI that could potentially aid in its clinical diagnosis. We further studied whether the impact of antibiotic usage on gut microbiome could be decoupled and distinguished from the gut dysbiosis observed after *C. difficile* active infection development.

We assembled the microbiome metagenomic datasets from the cohorts of three groups of human subjects from ten published studies: CDI+ cases, CDI- controls, and CDI- subjects but taking antibiotics. We aimed to develop predictive models for distinguishing these groups. The cohorts of CDI+ cases and CDI- controls also comprise those involved in both allogenic and non-allogenic FMT studies. We devised a hybrid species/function approach to determining the taxonomic profiles using the corresponding functional profiles of the microbiome samples. Using these functionally informed taxonomic profiles, we deployed logistic regression (LR) models that showed high prediction accuracy on distinguishing CDI+ cases from CDI- controls. These results suggest that the functional profile of the gut microbiome is a strong indicator of CDI. Furthermore, they indicate that the integration of metagenome sequencing data and machine learning offers not only an adequately reliable approach to CDI detection but also provides insights about factors and determinants of CDI etiology.

## Results

We collected the metagenomic sequencing data from ten previously published human gut microbiome studies (see Table 2 for details), acquired from 197 human subjects including 88 samples from 73 CDI+ patients (as cases) and 203 longitudinal samples from 94 CDI- individuals as well as 88 longitudinal samples from 30 healthy individuals who volunteered to take antibiotics (as controls). In all of these studies, the metagenomic sequences were generated using Illumina sequencers with reads length of 100bp. We used these data to test our hypothesis that the fecal microbiome in particular, the collective microbial functional dynamics, is predictive of *C. difficile* infection status of the host.

**Table 2.** Details on the data sources and sample size used in this study. CDI stands for *C. diff* infection, and MS stands for metabolic syndrome disorder.

Study	Accession	Clinical status		Intervention			Donors		Controls	
		Condition	Patients	type	pre [samples per subject]	post [samples per subject]	number	samples	number	samples
Smillie et al. 2018	PRJEB23524	CDI	19	FMT	19 [1 pre-FMT]	48 [1 to 4 post-FMT]	4	12		
Watson et al. 2021	PRJNA701961	CDI	10	FMT	19 [2 pre-FMT]	51 [4 to 9 post-FMT]	2	39		
Podlesny et al. 2021	PRJEB39023	CDI	8	FMT	8 [1 pre-FMT]	11 [1 to 2 post-FMT]	8	8		
Kim et al. 2020	PRJEB35738/PRJEB33013	CDI	26		26	26			60	60
Milani et al.2016	PRJNA297269	CDI	5						5 CDI-ABX+	5
			5						5 CDI-ABX-	5
Duan et al. 2020	PRJNA591064	CDI	5		5				4 CDnD	4
									5 CDnC	5
Lee et al. 2017	PRJNA353655	healthy		FMT	2 [1 pre-FMT]	4 [2 post-FMT]	2	4		
Li et al. 2016	PRJEB12357	MS	5	FMT	5 [1 pre-FMT]	20 [4 post-FMT]	3	5	5	5
		healthy		self-FMT	5 [1 pre-FMT]	20 [4 post-FMT]				
Raymond et al. 2016	PRJEB8094	Healthy		Antibiotics	18	36			5	15
Palleja et al. 2018	ERP022986	Healthy		Antibiotics	12	43				

## *C. difficile* is not more abundant in the gut microbiome of the CDI+ cases than those of the controls

**Figure 1.**
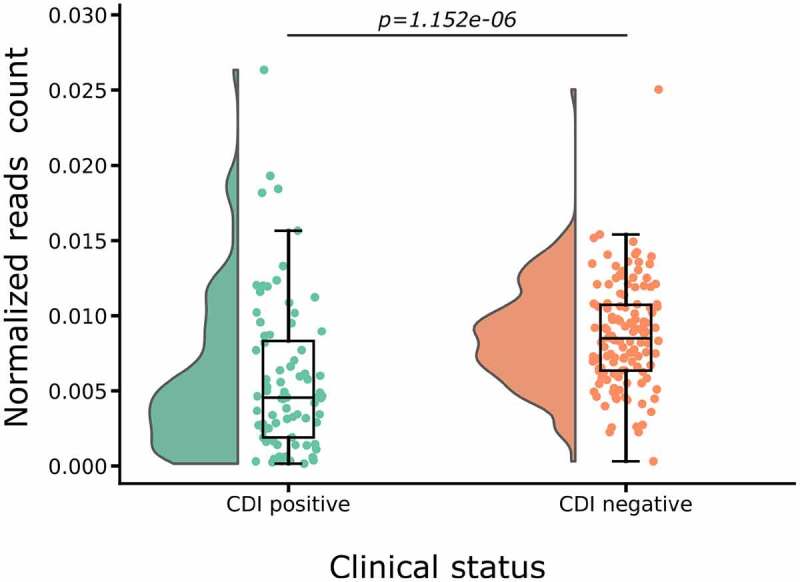
***C. difficile* strains abundance, estimated by the normalized read count of** *C. difficile* **genomes in the gut microbiome samples from CDI+ and CDI- individuals, respectively**. In the figure, each dot represents the normalized read count in a specific sample. The normalized read count was computed by counting all reads mapped to the 87 *C. difficile* genomes, and then normalized by the total number of reads in the microbiome sample. These results encompass samples from the 6 CDI cohorts analyzed in this study.

Previous studies have shown that *C. difficile* alters the gut microbiome to favor its growth and proliferation.^25,37^ We first determined the abundances of *C. difficile* in the gut microbiome of CDI+ patients versus CDI- controls from all the 6 CDI cohort studies. We assembled a total of 87 *C. difficile* genomes (see supplementary materials Table S9 for the list) and then estimated the *C. difficile* abundance in a microbiome sample based on the normalized count of reads that were mapped to these genomes. As shown in Figure 1, the normalized read count of *C. difficile* is $8.713\times 10^{-3}\pm 3.040\times 10^{-4}$ in the CDI- cohort, which is significantly greater than $5.797\times 10^{-3}\pm 5.715\times 10^{-4}$ in the CDI+ cohorts ($p-value=1.152\times 10^{-6}$ by Mann–Whitney U-test). This result is somewhat surprising but could also be a result of antibiotic treatment of the herein studied CDI+ patients. However, the normalized read count was significantly less in ABX+ group compared to the CDI+ patients, see Supplementary Figure S1. Nevertheless, this result indicates that simply profiling the abundance of *C. difficile* genomes in gut microbiome may not offer any predictive power for CDI.

## Species/function profiling of gut microbiome in CDI+ vs CDI- samples

### Model formalism

Sampling a microbial community for metagenomic sequencing analysis means collecting a mixture of DNA from different organisms (bacteria, archaea, eukaryotic cells, viral species, etc.) at different levels of abundances and taxonomic diversity. The uneven abundance problem in microbial samples exacerbates the risk of sampling high abundant microbial community members and missing the least abundant species. In addition, the current taxonomic profiling methods use some minimum detection thresholds and thus may further exclude less abundant or rare microbial members that could provide vital and critical relevant information, especially in relation to human health and diseases.

**Figure 2.**
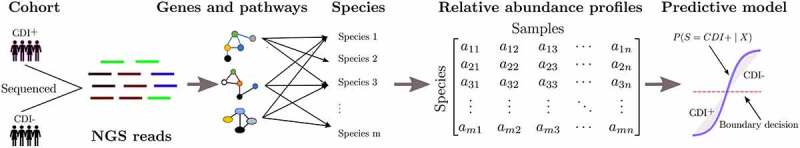
**The computational approach for species/function profiling of microbiome samples and construction of machine learning models for predicting the *C. difficile* active infection of the host**. This species/function hybrid approach aims at identifying microbial species/strains contributing to the observed functional profiles. We used the HUMAnN3 pipeline that analyzes the next-generation sequencing (NGS) reads from the metagenomic samples and reports the genes and functional profiles, from which the species abundances are inferred and then used for subsequent statistical and machine learning analyses.

In this study, we investigated the hypothesis that the functional profile of the gut microbiome from an individual (host) indicates the clinical status of the host with regard to CDI. We developed a hybrid approach to the species/function profiling of metagenomic samples from CDI+ cases and CDI- individuals. The hybrid approach starts with a conventional functional analysis identifying genes and biological pathways in metagenomic samples using HUMAnN3 software.^41^ Next, in contrast to the conventional reads coverage-based taxonomic profiling that relies either on the reference microbial genomes or the *de novo* assembled contigs/scaffolds, the presence and relative abundance of a taxon are defined by a linear sum of the contributions of all the genes in the sample that are also part of its genomic architecture (see details in Materials and methods section). This functional *genes-to-genomes* approach links functions to taxonomy and could capture the rare and less abundant taxa that are functionally important for the ecological dynamics of the microbial community.^42^ In the end, the inferred functionally informed taxonomic profiles were used as input features for machine learning prediction of CDI clinical status of the respective host. Figure 2 illustrates the workflow of the hybrid analysis.

## Gut microbiome of CDI+ cases is discernible from that of CDI- controls including those taking antibiotics

**Figure 3.**
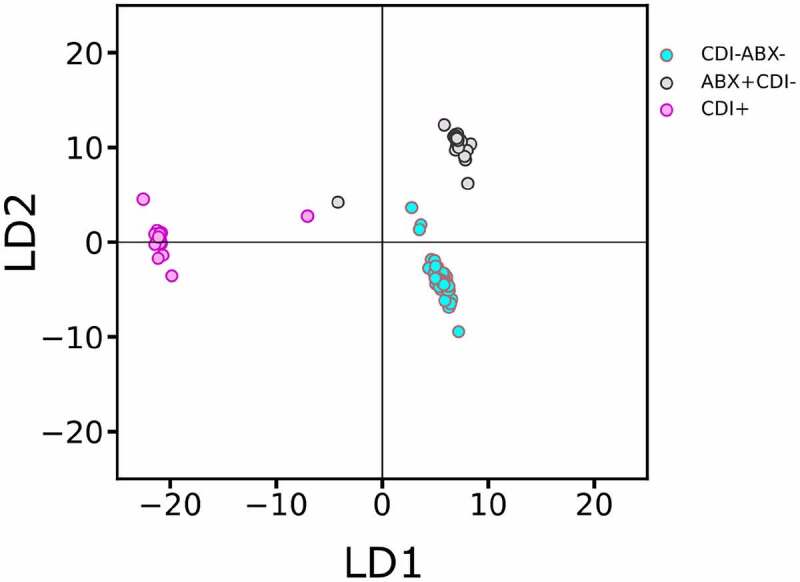
**Linear Discriminant Analysis (LDA) of three groups of samples: CDI+ in pink color, CDI-/ABX- cyan colored and CDI-/ABX+ in gray color, respectively**. Using the functionally informed taxonomic profiles, the three groups are separable and two linear discriminants explain the observed variation between the three groups where LD1 explains 78.41% and LD2 explains 21.59% of the observed variance.

We performed Linear Discriminant Analysis (LDA) on the species/function composition profiles inferred from the metagenomic samples of the three groups: CDI+ cases, CDI- but taking antibiotics (CDI-/ABX+), and CDI- not taking antibiotics (CDI-/ABX-), using the LDA class implemented in the python scikit-learn package.^43^ As shown in Figure 3, the three groups are separable on the basis of their respective functionally informed composition profiles. Specifically, CDI+ and CDI- (including CDI-/ABX+ and CDI-/ABX-) groups are clearly separated along the first linear discriminant (LD1 explaining 78.41% of the observed variance). These results indicate that the clinical status of CDI (CDI+ or CDI-) can be predicted from the functionally informed taxonomic profiles of the host’s fecal microbiome.

## Inferred microbial species/function profiles indicate *C. difficile* infection of the host

Using the aforementioned approach, a total of 642 microbial species were found to contribute to the distinguishable functional profiles of the three groups. We then built a logistic regression (LR) model to classify the host into these three groups using the microbial species/function profiles as the input features. We further used the logistic LASSO regression^44^ for selecting a small subset of features (i.e., microbial species), which are likely associated with CDI.

We evaluated the model in three different ways. First, we built the model using the samples from all studies, then assessed the model accuracy using a fivefold cross-validation (5-CV) approach, i.e., each time 80% of the data were used for training and the remaining 20% of the data were used for testing. The model prediction performance and accuracy were assessed by the area under the Receiver Operating Characteristic (ROC) curve (AUC) and the Matthew’s correlation coefficient (MCC) with a threshold of 0.50. The AUC evaluates the probability of correctly ranking pairs of negative and positive classes and provides the model performance across all thresholds but fails to account for potential class imbalance.^45^ We used MCC as an other evaluation metric that ranges between −1 and +1. MCC evaluates the classifier behavior where a higher MCC value indicates the model ability to make correct predictions on both positive and negative classes independent of the ratio of the classes or class label swapping.^46^ Thus, MCC provides a reliable means to evaluate the model performance where other evaluators (such as AUC and accuracy) might give overoptimistic performance results due to class imbalance in the data, for instance.

Next, we adopted a leave-one-study-out cross-validation strategy, where the samples in one study were used as the hold-out testing data, while the samples from the other studies were used to build and train the model, and the model’s prediction performance was assessed on the hold-out study.

Finally, we computed a log-odds score based on the probability $P(S_{i}=CDI+)$ reported for a specific sample $S_{i}$ by the model:

(1) $$\;\;Log\text{-}oddsscore=log\left(\frac{P(S_{i}=CDI+|X_{i})}{1-P(S_{i}=CDI+|X_{i})}\right)$$

where $X_{i}$ is the derived relative abundances of species contributing to the observed functional profile of the sample $S_{i}$.

**Figure 4.**
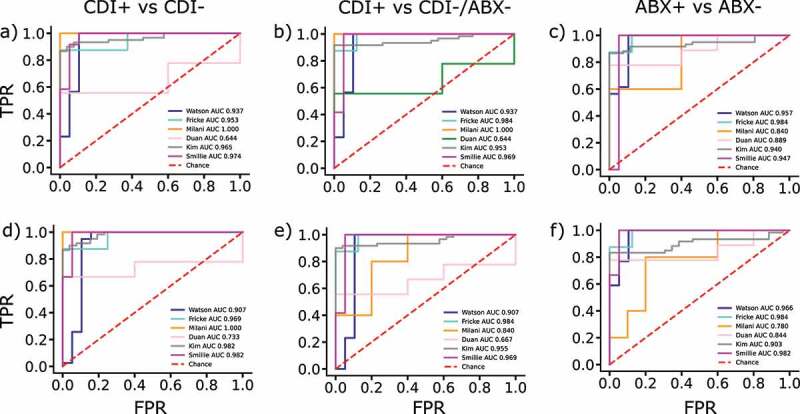
**CDI prediction based on the species/function profiles of the host’s fecal microbiome**. The leave-one-study-out validation results of the LR model (plots a-c) and the LASSO-LR model (plots d-f) and each column panel indicates the classes being evaluated: LR model for CDI+ vs CDI- in a), CDI+ vs CDI-/ABX- in b) and ABX+ vs ABX- in c). LASSO-LR predictions for CDI+ vs CDI- in d), CDI+ vs CDI-/ABX- in e) and ABX+ vs ABX- in f). In each plot, the AUC of a model trained on all the other cohorts and tested on the hold-out study is presented. Both LR and LASSO-LR models have similar performance, but the prediction of CDI is not as robust when dealing with diarrhea patients as in Duan et al study (lower AUC in all) or antibiotic usage as is the case in e) for the Milani study. FPR: false positive rate, TPR: true positive rate.

Figure 4 and supplementary Figures S2 and S3 show the predictive results of the logistic regression classifiers. Using all the 642 species, the LR model predicted CDI+ with an average AUC of 0.949 in 5-CV when we considered the two classes of CDI+ (as the *positive* class) and CDI-/ABX- (as the *negative* class). Given that CDI patients in these studies were treated with antibiotics or had prior antibiotic exposure, these predictions may be confounded by the impact of antibiotic treatment. To address this issue, we built an LR model considering the two classes of CDI+ (as the *positive* class), and the combined CDI-/ABX+ and CDI-/ABX-, denoted as CDI- (as the *negative* class). The new predictive model reached an average AUC of 0.919 in 5-CV, indicating a strong predictive power (see supplementary Figure S2 and Table S3). Using the leave-one-study-out cross validation, this model showed high accuracy (average AUC of 0.912) in predicting CDI+ vs CDI-. However, the model accuracy is lower for predicting CDI+ vs ABX+/CDI- samples in the Palleja et al. study^47^(accuracy = 80.0%) when the model was trained using the other samples from the other studies (see Table S3), perhaps due to the different antibiotic treatment used in this control study (a cocktail of three last resort antibiotics) versus the treatment used in the other study (to be discussed in detail later under Confounding impact of antibiotic treatment on CDI section).

**Figure 5.**
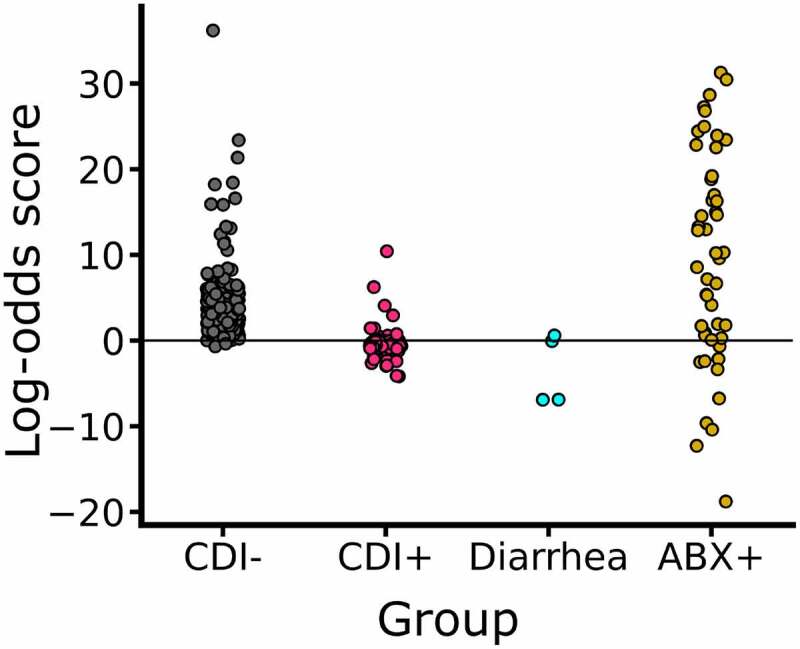
**CDI prediction based on a consortium of 21 species significantly associated with CDI**. The species were selected by the LASSO-LR algorithm (see text for details).

We further evaluated the predictive models built by using the logistic LASSO regression (LASSO-LR), which selects a small subset of features and thus is often more robust.^48^ The LASSO-LR model reached an average AUC of 0.951 in 5-CV on the classification between the two classes of CDI+ vs CDI- and an average AUC of 0.947 in 5-CV on the classification between the two classes of CDI+ vs CDI-/ABX- (Figure S2A and Figure S2B, respectively.) The accuracies of LASSO-LR models are comparable with those of the LR models, even though LASSO-LR selected only 21 (out of 642) species as input features, which indicates that only a small number of species in the gut microbial communities are sufficient to give a good prediction of CDI. Using these 21 species (see list in the supplementary Table TS4), we built an LR binary classifier and computed the log-odds scores of CDI+ patients, CD-/ABX-, and ABX+ subjects. As shown, in Figure 5, the log-odds score is mostly negative for CDI+ cases, while it is mostly positive for CDI- controls including those taking antibiotics (ABX+ group). The log-odds scores are even more negative in some of the diarrhea patients even though they are CDI- which may imply that the gut microbiome of CDI+ patients shares some features with those from patients of other gut diseases, Figure 5.

The leave-one-study-out cross-validation of the LASSO-LR models demonstrated high accuracies in predicting CDI cases and the quality of the predictions is in strong agreement with the input classes, as shown in Figure 4a-f and Table 1. We observed that the LASSO-LR models classifying CDI+ vs CDI- showed much lower prediction accuracy (MCC = 0.258) on the samples from the study of Duan et al.^19^ in the cross-sample validation evaluation. We also observed that the LASSO-LR model for classifying CDI+ vs CDI-/ABX- performed poorly on the samples from the studies of Duan et al.^19^ and Milani et al.,^49^ as illustrated in Table 1 and Table S3. Notably, the Duan et al. study included 5 CDI+ cases, 4 CDI- patients but with diarrhea symptoms, and 5 CDI- healthy controls, whereas the Milani et al. study included 5 CDI+ cases, 5 CDI- patients who had extra-intestinal infectious diseases and were taking antibiotics, and 5 CDI- patients who had extra-intestinal noninfectious diseases but were not taking antibiotics. We observed that the LASSO-LR models classifying CDI- vs CDI-/ABX- tended to predict the patients who had other gastrointestinal conditions and taking antibiotics as CDI cases, while the LASSO-LR models classifying CDI+ vs CDI-, which included the antibiotics-treated healthy controls in the negative class, showed enhanced predictive power of discerning CDI- patients taking antibiotics from the CDI+ cases, but showed poor predictive power on the patients with diarrhea symptoms from Duan et al. study (for details see Table 1 and supplementary materials Table S3). We also observed that if we hold out the CDI- diarrhea patients from the training sample, both the LR and LASSO-LR models improve slightly their predictive performance (see supplementary Figures S3, S4). On the other hand, the model accuracies are much higher when a broad range of samples from all studies are used for model construction, Figure S2. These results demonstrated the advantage of the meta-analysis performed here that covered a comprehensive set of negative controls (with various clinical conditions and/or treatment) for building an accurate predictive model.

**Table 1.** The performance of predictive models in the cross-sample validation.

	Watson	Fricke	Milani	Duan	Kim	Smillie
	**LASSO-LR CDI+ vs CDI-/ABX-**					
Accuracy	0.931	0.938	0.467	0.643	0.930	0.935
AUC	0.908	0.984	0.780	0.667	0.955	0.969
MCC	0.846	0.882	0.277	0.471	0.855	0.876
	**LASSO-LR CDI+ vs CDI-**					
Accuracy	0.914	0.875	1.000	0.643	0.919	0.935
AUC	0.907	0.969	1.000	0.733	0.982	0.982
MCC	0.808	0.750	1.00	0.258	0.809	0.876

## Confounding impact of antibiotic treatment on CDI

Given that CDI patients are generally treated with antibiotics, failure to consider the potential impact of antibiotics on CDI prediction may lead to the CDI prediction confounded with impact of the antibiotic treatment on the host gut microbiome. On the one hand, various studies have shown that antibiotic usage alters the composition and decreases the richness and diversity of the host gut microbiome.^47,50^ On the other hand, prolonged usage of antibiotics is a known risk factor for *C. difficile* infection.^30^ However, even though the mechanism behind the interplay between antibiotics and CDI is relatively known,^29^ factors distinguishing dysbiotic modulations due to antibiotic usage from CDI gut dysbiosis are not well understood.

Here, we attempted to evaluate the confounding impact of antibiotic treatment on CDI by assessing the predictive power of the LR models on three tasks: 1) the prediction of CDI independent of the antibiotic treatment (Figure 4a,d), 2) the prediction of antibiotic treatment regardless of CDI status (Figure 4c,f), and 3) the classification between CDI+ cases vs antibiotic treated individuals (ABX+; Figure S2 D). For task 2, CDI+ cases and ABX+ cases (taking and after taking antibiotics) form one class (the negative class), while CDI- and ABX- form the positive class. We used data from Palleja et al.,^47^ where healthy individuals were given a cocktail of the three last resort antibiotics (meropenem, gentamicin, and vancomycin) for 4 days and followed up to 180 days post-intervention. We also used data from the Raymond et al. study^50^ in which healthy individuals were administered a second-generation cephalosporin, cefprozil for 7 days and followed for up to 3 months.

We found that the LASSO-LR models can successfully predict CDI independent of the antibiotic treatment (task 1, with an average AUC of 0.951 and an MCC value of 0.810) and the antibiotic treatment regardless of CDI or not (task 2, with an average AUC of 0.886 and an MCC of 0.670), see supplementary figures S2A and S2C. Furthermore, we observed that despite the effect of antibiotics, CDI+ cases can be distinguished from ABX+ cases with high predictive accuracy (average AUC = 0.935; supplementary Figure S2 D). However, among the healthy individuals who took the cocktail of the three last resort antibiotics, half of them on day 4 of the antibiotic usage and 73% of them on day 4 post-usage were predicted as CDI+ (i.e., the false positives; see Supplementary materials, Table S3). On days 42 and 180, the functional and species profiles of their gut microbiome moved closer to those samples where the hosts did not use antibiotics and the samples were predicted as true CDI-. These results suggest that strong usage of antibiotics may create a microbiome environment (in terms of the functional and species composition) that somewhat resembles that of CDI. This seems to be consistent with the previous study that indicated that the risk for CDI is elevated during the prolonged use of antibiotics or within a month of its usage.^30^

## Assessing the effectiveness of fecal microbiota transplantation (FMT)

Fecal microbiota transplantation (FMT) is recognized as an effective therapeutic option for CDI treatment.^51,52^ We evaluated the extent to which our model for CDI prediction can be exploited for assessing the effectiveness of FMT based on the post-FMT gut microbiome samples from the patients. We utilized the log-odds score computed from the output of the CDI prediction model (Equation 1) to measure the likelihood of an effective FMT: the low (negative) score on the post-FMT sample indicates it remains to be likely *C. difficile* infected, and thus the FMT is less effective, whereas a high (positive) score indicates the sample to become unlikely *C. diff*icile infected, and thus the FMT is more effective.

We studied the previously published post-FMT data acquired after four types of FMT: 1) an *autologous* FMT,^53^ where the microbiota from a healthy donor was transplanted to the same subject; 2) a *non-allogenic* FMT, where the microbiota from a single healthy donor was transplanted to two healthy subjects who have no known underlying clinical conditions;^54^ 3) an *allogenic* FMT, where the microbiota from three separate healthy donors were transplanted to five recipients with metabolic syndrome (MS);^53^ and 4) an *allogenic* CDI FMT where ten and nineteen CDI patients received fecal microbiota transplantation from two to four healthy donors,^17,55^ respectively.

**Figure 6.**
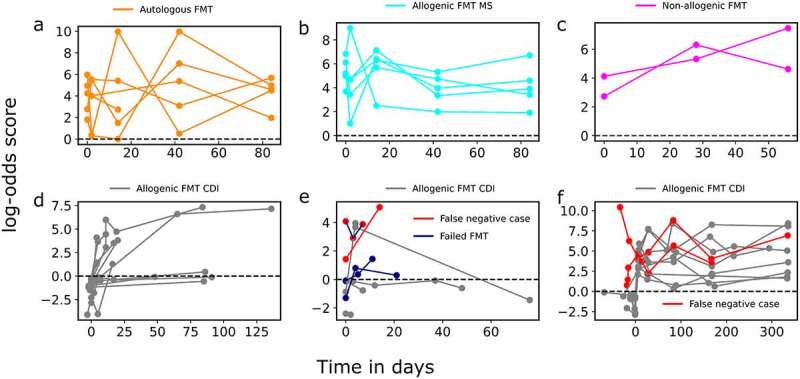
**Evaluation of FMT effectiveness based on CDI prediction over post-FMT host microbiome data**. A log-odds score is computed from the probability output by the LASSO-LR binary classifier, where CDI+ cases are labeled as the class 0 and CDI- controls are labeled as the class 1. Thus, the negative scores indicate likely CDI+ while the positive scores indicate CDI-. Each connected line corresponds to one subject receiving FMT. Figures a-c serve as the positive control for evaluating CDI FMT. Figure a) shows log-odds score for 5 healthy individuals who received their own fecal stool (autologous FMT) and b) indicates scores for 5 MS patients treated by FMT and c) includes results from 2 healthy individuals who received fecal stool from the same donor. Figures d and e indicate scores of the Smillie cohort where d) shows 11 patients who showed a gradual increase in their scores post-FMT while e) indicates 2 individuals that were misclassified (red color), 2 unsuccessful FMT (blue color) and 4 others who showed a decline in their scores post-FMT. Figure f) shows 10 rCDI patients from the Watson et al. study and indicates that despite fluctuations in their log-odds scores, these rCDI patients remained consistently in remission (red color indicates 2 misclassified cases).

As shown in Figure 6a-c, the LASSO-LR model gives a reliable prediction (with the positive log-odds score) for all the CDI- subjects (Types 1, 2 and 3; see above) using their pre-FMT and post-FMT gut microbiome samples. Figures 6d and 6e show the change of the log-odds scores on the pre-FMT and post-FMT samples from the CDI+ patients receiving allogenic CDI FMT (Type 4). Figure 6d shows the subjects from the Smillie et al. study,^17^ which studied the post-FMT samples from the CDI+ patients in a short period of time (the longest up to 135 days), and Figure 6f showed the subjects from the Watson et al. study,^55^ which followed the CDI+ patients in the long term (up to 336 days) after they received FMT. We observed that the LASSO-LR model miss-classified two CDI+ patients in these two cohorts, respectively, even though the overall prediction accuracy is satisfactory (i.e., 93.5% and 93.1%, respectively). Interestingly, for all these CDI+ patients, the log-odds scores on their post-FMT samples are higher comparing with their pre-FMT samples in the Watson et al. study and in the Smillie data except 4 patients. Among these cases, three FMT cases were reported as failure in the Smillie study (highlighted in orange and marked with a star in Figure 6e), among which one has a relatively small log-odds decrease compared with the other cases, though this individual is also predicted a false negative by the LASSO-LR model (highlighted in blue and marked with a red head Figure 6e). We observed that two cases who shortly after FMT procedure showed improvement (increase in their log-odds score), their log-odds scores have decreased 45 and 75 days post-FMT, respectively, Figure 6e). After all, the increase of the rate of change of log-odds score after FMT is gradual shortly after the intervention (FMT procedure), (see Figure 6d) and consistent over the long period of follow-up (Figure 6f), implying the improvement of the clinical condition is subtle in the short term (Figure 6d) but steady and significant in the long term (Figure 6f) i.e., fluctuations in log-odds remain above 0 consistent with CDI- profiles.

## Functional profiles of the gut microbiome from CDI+ patients elucidate pathways indicating potential *C. difficile* pathogenesis and colonization

The analyses of the functional profiles of CDI+, CDI-/ABX-, and CDI-/ABX+ samples revealed 603 pathways with consistent abundance patterns across samples. Using a *p-value* threshold of $1\times 10^{-4}$ (2-tail t-test), we found 271 pathways whose abundances are significantly different between CDI-/ABX- and CDI+ (for the full list, see Supplementary table TS5). Among these pathways, 42 are significantly different between CDI-/ABX- vs CDI-/ABX+, while 204 are significantly different between CDI-/ABX+ and CDI+. We also observed that 14 pathways exhibit a significant difference across the three categories (CDI-/ABX- vs CDI-/ABX+ vs CDI+ (by pair-wise 2-tail t-test)) whereas 29 pathways show no significant difference between CDI+ and ABX+ groups. Among the remaining 332 pathways that were not found significantly different between CDI+ and CDI-/ABX- by our criteria, 33 pathways were found to show the significant impact in ABX+ group compared to the other two groups, CDI-/ABX- and CDI+.

**Figure 7.**
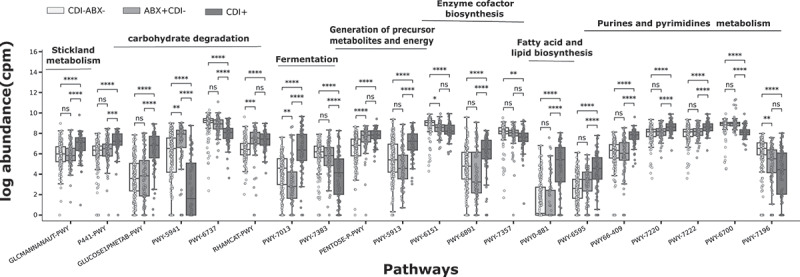
**Pathways significantly different in CDI+ vs CDI- samples**. These pathways are selected by a LASSO-LR model to predict with high accuracy CDI+ vs CDI-. In the figure, using a 2-tail t-test, ”ns” indicates a no significant statistical difference between the means of the two groups, **** indicates a statistical significance with a p-value $\leq 1\times 10^{-4}$, *** indicates a p-value $\leq 1\times 10^{-3}$ and ** a p-value $\leq 1\times 10^{-2}$.

Consistent with previous studies,^37,56,57^ we observed an elevated presence of pathways involved in central carbon metabolism (GLUCOSE1PMETAB-PWY, PENTOSE-P-PWY, GALACTARDEG-PWY, P461-PWY, etc.) and in nucleotides and amino acid metabolism especially the Stickland metabolism (such as GLCMANNANAUT-PWY and ARGDEG-PWY) and purines *de novo* biosynthesis (such as PWY-7220, PWY-7222) in CDI+ than in CDI- samples (Figure 7 and supplementary Figure S6-7). On the other hand, we observed that pathways involved in complex sugar metabolism (such as PWY-6737) are highly abundant in CDI- compared to CDI+ samples.

Our results shed light on the dynamics in the gut ecosystem that potentially facilitate CDI pathogenesis. We observed the increased presence of pathways involved in fatty acid biosynthesis, especially lipopolysaccharides (LPS) (such as LPSSYN-PWY, PHOSLIPSYN-PWY, NAGLIPASYN-PWY) in CDI+ compared to CDI-. Bacterial LPS are complex glycolipids located in the outer membrane of all gram-negative bacteria and are essential for their growth and viability.^58^ Proinflammatory LPS are known to cause or contribute to inflammation-related diseases.^59^ Both *Klebsiella pneumoniae* and *Escherichia coli*, two of the identified 21 bacteria predominant in CDI+ harbor the pathways associated with LPS. In addition, pathways involved in the heme biosynthesis and the electron transfer cofactors are significantly increased in CDI+ cases (see Figure S6), which paints the picture of a molecular ecosystem showing signs of inflammation and oxidative stress. Antibiotic impact on gut microbiome could also be mediated by this lipid-induced inflammation. We observed a moderate increase in abundance in pathways of fatty acid biosynthesis (Figure S6). The oxidative stress and inflammation of the protective layer may lead to the release of the host heme, which *C. difficile* takes in its defense system to protect itself against antibiotic activities.^60^ This suggests that *C. difficile* colonization may be enabled by other bacteria producing an excess of free heme, which is known to cause the oxidative stress and tissue injury.^61^ These findings indicate that the severity and manifestations of CDI result from interspecies interactions. In fact, each of the 21 species that were identified in this study contributes to some or all the observed significantly differential pathways (Table S7). The complete summary of the pathways enriched in both CDI+ and CDI- samples is provided in the supplementary Figure S6-7 and Table S5.

## Discussion

Our study showed the great potential of fecal microbiome samples for CDI detection. Specifically, the predictive model exploiting the microbial functional profile or the species composition contributing to the functional profile can accurately detect CDI. We identified 21 species that have robust prediction of CDI. Among these selected species, there are species that are known commensals such as *Faecalibacterium prausnitzii* and *Bacteroides thetaiotaomicron*^62^ and species that are known to co-colonize with *C. difficile* such as *Klebsiella pneumoniae*,^63^ and species that are reported to provide some protection against CDI such as *Prevotella copri* and *Ruminococcus gnavus*.^64^

However, despite high prediction accuracy, the model classifies some CDI- subjects taking antibiotics as CDI+ cases, in particular those taking a cocktail of the three last resort antibiotics. In these cases, the antibiotic impact is significant 4 days after cessation: the species/functional profiles of these gut microbiome samples are similar to those of the CDI+ cases. These results suggest that the impact of antibiotics on potential CDI development could be significant during the usage and shortly after the treatment, which is consistent with previous studies.^30^ In addition, despite the overall high accuracy, our model misclassifies some CDI- patients (according to stool tests) who suffered from diarrhea as CDI+. On the one hand, this may be due to the lack of sufficient training samples (we have four such patients in total) for our model to learn the specific functional profiles of the microbiome samples in this group. On the other hand, it is possible that the model did not learn the representation of specific disease stages because we have a relatively small training dataset (369 samples in total including both positive and negative samples), while the CDI+ patients were at a different stage and thus have different gut microbiome conditions. In fact, previous studies have encountered this challenge. For instance, Manor et al. in their recent study evaluated the association of microbiome with various host phenotypes, and reported that the host-microbiome associations are predominantly context dependent and microbial composition-specific.^20^ Other meta-analyses investigating how microbiome relates to the human health also reported that the microbial functional markers are associated with a wide range of diseases with only a particular subset of them associated with specific diseases.^21,65^ These studies and many others have mostly used only one-time samples from participants, and thus lack the added value of longitudinal data, which raises the question of whether a detected host-microbiome association is a true signature of a disease or the signal of a shared response or a nonspecific trait related to the disease.^66^ To address this issue, here, we combined both longitudinal and space-resolved samples and focused on the species that contribute to the distinctive functional profiles between the two classes of samples. This approach may be applied to the study of the association between human microbiome and other clinical conditions affecting humans.

Ideally, a comprehensive functional analysis of microbial communities allows us to understand the ecological dynamics and to establish the nature of the host–microbiome relationship. However, such analysis would mandate the integration of various meta-omics data from metagenomics, metatranscriptomics, metaproteomics, and metabolomics augmented with other auxiliary metadata. Unfortunately, this level of analysis is currently in its infancy and remains expensive, and as a result, the data are scarce. Nonetheless, the functional analysis of microbial communities based solely on metagenomics data provides a proxy measure of the potential functional profile of the sampled microbial community. Intuitively, if any significant alteration in the functional capacity of a microbial community potentially results in meaningful biological implications on the equilibrium and the stability of the microbiome, such perturbation would lead to the change of the host phenotype (i.e., the clinical condition). Therefore, the adequate analyses of metagenomic data acquired from clinical samples^67–73^ may lead to the discovery of microbiome markers that give hints on novel diagnostic and therapeutic approaches, as we demonstrated in our study.

## Materials and methods

### Data collection and study population

All the data used in this study are publicly available and were downloaded from the NCBI SRA repository databases. We collected the whole metagenome sequencing (WGS) data spanning CDI patients (CDI+), CDI- individuals not taking antibiotics (CDI-/ABX-), and healthy individuals who volunteered to take antibiotics (CDI-/ABX+). We summarized the source and description of the datasets in Table 2. We used the clinical data from Smillie et al.,^17^ including 19 rCDI patients aged between 7 and 90 years enrolled in an FMT treatment study involving 3 healthy donors. We also used data from Duan et al.^19^ encompassing 5 CDI patients, 4 CDI negative diarrhea patients (CDnD) and 5 CDI negative controls (CDnC). Furthermore, we used the data from Milani et al.^49^ In this study, Milani et al. evaluated the composition of gut microbiome from 5 CDI+ elderly patients, 5 CDI- patients with extra-intestinal infectious diseases taking antibiotics, and 5 CDI- patients suffering from other extra-intestinal noninfectious diseases. We also used data from Kim et al.^18^ comprising 26 CDI+ patients and 60 healthy individuals. We also included the rCDI patients previously studied by Watson et al.^55^ This cohort included 10 rCDI patients who were treated with antibiotics 4 times per day for a period of 10 days prior to undergoing the FMT procedure. Watson et al. aimed at evaluating the drivers of the human gut colonization post-FMT and presented longitudinal data for the ten rCDI cases and for two donors sampled up to a year post-FMT.^55^ Another FMT dataset we used comes from Podlesny and Fricke,^74^ which studied the dynamics of strain engraftment post-FMT. The study involved eight rCDI cases who have had at least three CDI recurrences and received at least three courses of antibiotics prior to FMT treatment. These patients received fecal transplant from eight related CDI- donors. The CDI patients in these studies were treated with antibiotics or had prior exposure to antibiotics. To study the potential confounding impact of antibiotics on gut microbiome, we collected data from Palleja et al., which studied the impact of antibiotics in 12 healthy individuals who volunteered to take a cocktail of 3 last resort antibiotics (meropenem, gentamicin, and vancomycin) for 4 days,^47^ and collected gut microbiome data up to 180 days after the intervention. Finally, we collected the data from another intervention study that used a second-generation cephalosporin, cefprozil on 18 healthy volunteers for 7 days, and collected the gut microbiome data up to 90 days.^50^ Finally, we also studied cohorts from two different non-CDI FMT studies. Data from Li et al.^53^ included fecal metagenomes from five metabolic syndrome patients who received fecal transplant from three lean healthy donors. This cohort also included five individuals who received an autologous FMT [i.e., the same individual receives fecal transplant from their own stool]. Lastly, we collected data from Lee et al., who studied microbiota colonization in FMT using two unrelated healthy individuals who received fecal transplant from the same donor.^54^ A detailed description of the studied cohorts is provided in supplementary Table TS10.

### Data preprocessing and preparation

We only considered and used the whole metagenome sequencing paired-end data generated using Illumina sequencing technology. For each study, NGS metagenomic reads were downloaded from the NCBI SRA repository using the SRA Toolkit *fastq-dump* command. The quality control check was done using FastQC software^75^ and reads were trimmed when necessary using Trimmomatic-0.39 using the following parameters LEADING:3 TRAILING:3 MINLEN:36 along with the corresponding adapters used for each specific study.^76^

### Estimation of the abundance of non-redundant C.difficile genomes

We collected the complete genomes of 87 *C. difficile* strains (supplementary materials, Table S9) from the NCBI database. We excluded other genomes that were partially assembled. *Bowtie2* with the default settings was used for reads mapping,^77^ and *samtools* was used to count the number of mapped and unmapped short reads to each genome in an input sample. The number of mapped reads to the *C. difficile* genomes was normalized by the total number of all reads as in Equation 2:

(2) $$c=\frac{\sum_{i}^{N}g_{i}}{\sum_{I}^{N}g_{i}+U_{i}}$$

where c is the normalized reads count, $g_{i}$ is the number of reads mapped to genome i, N is the number of genomes, and $U_{i}$ is the number of unmapped short reads in the sample. This normalized read count was then used to compare CDI+ cases and CDI- controls and those taking antibiotics.

### Functional and taxonomic profiling

The functional profiling was done using the high quality reads from each study. We used the software HUMAnN3.^41^ This functional profiler was selected because of its ability to stratify community functional profiles according to contributing species and it has shown high accuracy in detecting and quantifying species contributions to community functional profiles compared to others. HUMAnN3 profiles genes, pathways, and modules from metagenomes using native UniRef90 annotations from ChocoPhlAn species pangenomes.^78^ HUMAnN3 then reports the relative abundance of each gene and biopathway detected in the community and provides a stratified contribution from the species profiled using MetaPhlAn3.^41^ This allows us to use this stratification to determine the relative abundances of the contributing species in the community by summing up all the normalized relative abundances (in copies per million (cpm) units) of the pathways that the respective species contributes to the community.

### Calculation of the relative abundance of the species in functionally informed taxonomic profiles

The relative abundance of the species derived from the functionally informed profiles was calculated and determined as a linear sum of the species contributions to the various pathways detected in the functional profile of the sample: $f(species)=\sum_{j=1}(p_{j}|p_{j}\in P)$ where P is the set of all pathways detected in the sample and $p_{j}$ denotes any pathway that species j contributes to. In a more formal way, for a species i, its functionally informed relative abundance is calculated as indicated in Equation 3:

(3) $$f_{i}=\sum_{p}^{P}a_{ip}$$

where $a_{ip}$ is the fraction of reads assigned to species i and to the pathway p. It is important to note that functionally informed profiles are reliant on the functional annotations of the microorganisms. Additionally, there are a few limitations to this approach. First, the implementation of this method in this work does not take into account that genes may be contributing to various molecular pathways which could inflate the estimated abundance of the contributing taxon. Secondly, its implementation here is also based on HUMAnN3 which uses a reference-based approach for functional profiling. Therefore, the findings will be reliant on the quality and quantity of the used references.

### Comparison to other taxonomic profiling approaches

We conducted a benchmarking comparison between our method with Metaphlan3^41^ and Kraken2.^79^ Kraken2 is a profiler that uses pattern-match or exact match of *k-mers* facilitated by classification algorithms, while Metaphlan3 is a homology-based approach that uses specific gene markers for inferring taxonomic abundances. We compared these approaches on real data from two cohorts, Smillie et al.^17^ and Li et al.^53^ datasets. We further used the Spearman correlation metric to compare their estimates. The results indicated that the functionally informed taxonomic profiles correlate better with Metaphlan3 profiles (between 0.70 and 0.80) compared to Kraken2 (≤0.60), as shown in Supplementary Figure S8.

### Integration of taxonomic and functional profiles from multiple studies

Samples from different cohorts were joined using the *humann_join_tables* command of the HUMAnN3 pipeline. Ultimately, the integrated data were used as input for downstream machine learning classification and predictive analyses.

## Machine learning analyses

### Linear discriminant analysis

To evaluate microbial feature distinction between CDI patients and CDI- negative controls including those taking antibiotics, data were divided in three categories, namely, CDI+, CDI-, and ABX+ groups. To detect potential batch effect and technical variations due to specific factors such as sample handling or geography, data were categorized based on that specific variable (geography, sample preparation (cohort), etc.). Then, we use the *LDA* module in the sk-learn python package^43^ with *n_components* set to 2 to perform the LDA on the integrated data. The variation among the data was subsequently measured by the sum of the variations explained by the two linear discriminants.

### Regression and classification analyses

In order to learn the relationship between the fecal metagenome and CDI, we used a binary logistic regression (LR) model. In this supervised learning task, we considered N input/output pairs of training instances, $\left\{(x^{\left(i\right)},y^{\left(i\right)})\right\}$, for i=1,2,…,N, where $x^{\left(i\right)}\in R^{m}$ is an m-dimension feature vector representing the species relative abundances, while $y^{\left(i\right)}\in \{0,1\}$ is a class label, where a sample is labeled 0 (e.g., if it is CDI+) or 1 (e.g., if it is CDI-). We used customized python scripts to build an LR classifier that outputs p(y|x,θ), where θ represents parameters of the LR model (i.e., the weights w and the intercept b). Further, we used logistic LASSO regression (LASSO-LR) for feature selection and identification of the species associated with the host condition (e.g., CDI+ or CDI-). The samples were grouped into classes depending on the task of the binary classification, e.g., for the model to classify CDI+ vs CDI-, the CDI- class consisted of samples from the CDI-/ABX- and the CDI-/ABX+ human subjects; for the model to classify ABX+ vs ABX-, the ABX+ class included samples from the CDI+ and the CDI-/ABX+ subjects, the ABX- class consisted of only samples from the CDI-/ABX- subjects. We used LASSO-LR using the *LogisticRegressionCV* module in the sk-learn and Yellowbrick^80^ python packages.^43^ Hyper-parameters tuning and optimization were done using *gridSearchCV* with a 5-CV. The solver *liblinear* with the *l1* penalty was the best optimizer and was used for the LASSO-LR model with a 5-CV and the maximum number of iterations set to 10000. This optimizer was then used along with the *selectFromModel* module to select the non-zero coefficient microbial features (species or pathways) for further prediction and classification analyses. The customized scripts should be accessible at https://github.com/Enzabe/GiMicro.

### Comparison of functional profiles

For the comparison of functional profiles, the relative abundance of each detected pathway was averaged in the integrated data based on CDI clinical status. We used a 2-tail t-test to determine the difference between functional abundance in diseased vs non-diseased gut microbiomes using the python *scipy.stats* module. We classified the different pathways into biological processes based on the MetaCyc database of metabolic pathways and enzymes and the BioCyc collection of Pathway/Genome Databases.^81^

## Supplementary Material

Supplemental Material

## Data Availability

All the data used in this study are publicly available, and their accession numbers are provided in Table 2.
