# Supplementary figures and images for: Functional profile of host microbiome indicates Clostridioides difficile infection
Source: Gut Microbes. 2022 Oct 26;14(1):2135963. doi: 10.1080/19490976.2022.2135963 (PMC9621045; doi:10.1080/19490976.2022.2135963)

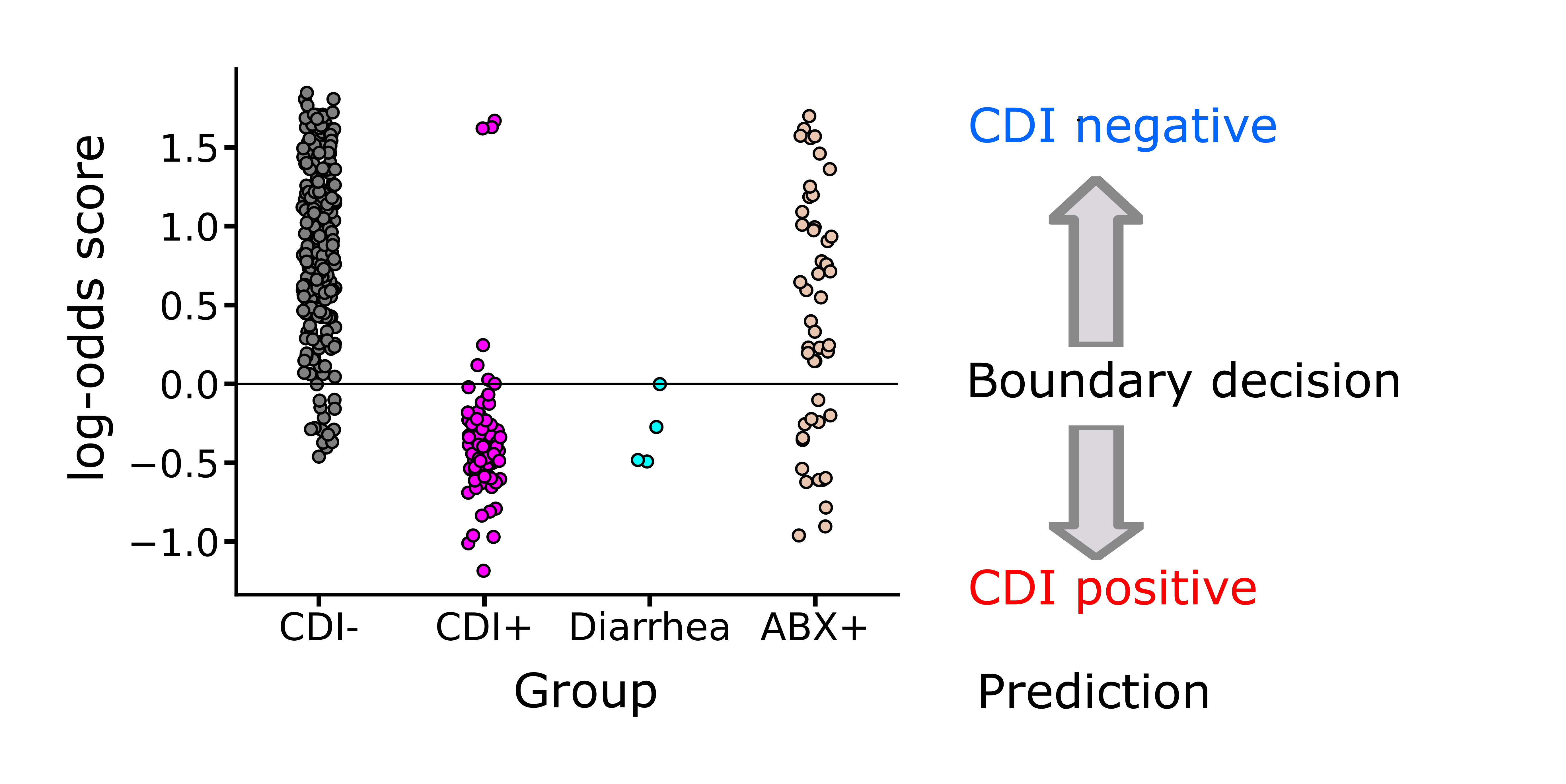

Supplement: Supplemental Material [file KGMI_A_2135963_SM6284.zip › SupplFigure-FigS5.tiff]
